# Eosinophilic esophagitis

**DOI:** 10.1186/1710-1492-7-S1-S8

**Published:** 2011-11-10

**Authors:** Stuart Carr, Wade Watson

**Affiliations:** 1 University of Alberta, Division of Clinical Immunology & Allergy, Edmonton, Alberta, Canada; 2 Dalhousie University, Division of Allergy, IWK Health Centre, Halifax, Nova Scotia, Canada

## Abstract

Eosinophilic esophagitis (EoE) is an atopic condition of the esophagus that has become increasingly recognized over the last decade. Diagnosis of the disorder is dependent on the patient’s clinical manifestations and histologic findings on esophageal mucosal biopsies. Patients with eosinophilic esophagitis should be referred to both an allergist and gastroenterologist for optimal management, which may include dietary modifications, pharmacologic agents such as corticosteroids, leukotriene modifiers and biologics as well as mechanical dilatation of the esophagus. The epidemiology, pathophysiology, diagnosis, treatment, and prognosis of EoE are discussed in this review.

## Introduction

Eosinophilic esophagitis (EoE) is an atopic inflammatory disease of the esophagus that has become increasingly recognized in children and adults over the last decade. The disorder is sometimes referred to as “asthma of the esophagus” given that it shares many clinical and pathophysiologic characteristics with asthma [1].

Eosinophils are typically present throughout the gastrointestinal tract since it is continuously exposed to foods, environmental allergens, toxins, and pathogens. Interestingly, in healthy individuals, the esophagus is unique in that eosinophils are generally absent. In EoE, however, eosinophils infiltrate the esophagus, contributing to tissue damage and chronic inflammation. EoE is defined as a clinicopathologic disorder characterized by >15 eosinophils per high power field [HPF] in one or more esophageal biopsy specimens and the absence of pathologic gastrointestinal reflux disease (GERD) (as evidenced by a normal pH monitoring study or lack of response to adequate acid-suppression therapy) [2].

The increasing number of recognized cases of EoE has resulted in a dramatic expansion of the medical literature surrounding the disease. This article provides a practical overview of recent literature surrounding the epidemiology, pathophysiology, diagnosis, treatment, and prognosis of EoE.

## Epidemiology

Given the poor awareness and recognition of the disease in the past, the epidemiology of EoE is still unclear. In children/adolescents up to 19 years of age, current prevalence estimates range from 1 to 4 per 10,000 persons [3]. Recent literature suggests that the prevalence of EoE is increasing [4]. However, there is debate as to whether the new cases of EoE being diagnosed represent a true increase in prevalence or rather increased recognition of latent disease. Furthermore, esophageal endoscopic biopsies are currently required to establish the diagnosis of EoE and, therefore, variations in endoscopy practices may bias the results of epidemiologic studies. For example, some studies suggest that when correcting for the number of endoscopies/biopsies being performed, the perceived increase in the prevalence of EoE may not be as dramatic as originally postulated [5].

Evidence also suggests that there is both ethnic and gender variation in the prevalence of EoE, with the majority of cases reported in Caucasian males. However, this finding is also uncertain since this is the patient population that has been most extensively studied [6,7]. Further population-based, epidemiological studies are needed to investigate the true prevalence of EoE, particularly in the adult population.

## Pathophysiology

Although the pathogenesis of EoE remains unclear, evidence suggests that the disease is associated with T helper cell (Th)-2 type immune responses, which are typical of other atopic conditions. In particular, elevated levels of the Th2 cytokines interleukin (IL)-4, IL-5, and IL-13, as well as mast cells, have been found in the esophagus of EoE patients [8-10]. These cytokines appear to play an important role in the activation and recruitment of eosinophils to the esophagus. Furthermore, there is evidence suggesting a genetic predisposition for the disease since the gene for eotaxin-3 – a chemokine involved in promoting eosinophil accumulation and adhesion – has been found to be overexpressed in patients with EoE [9].

EoE is also believed to be a mixed immunoglobulin (Ig)E- and non-IgE–mediated allergic response to food and environmental allergens [11,12]. IgE-mediated reactions are immediate hypersensitivity responses that usually occur within minutes after exposure to an allergen. Non-IgE mediated allergic disorders are characterized by a delayed onset (hours or days after exposure to the antigen) and potentially more chronic symptoms. The majority of patients with EoE have been found to have positive skin prick tests (which detect IgE-mediated reactions) and atopy patch tests (which may identify non-IgE-mediated reactions) to foods and/or aeroallergens. However, whether or not sensitization (positive testing) to these allergens establishes a causal role in EoE remains unclear.

## Diagnosis and investigations

Since the physical examination of patients with EoE is often unrevealing, the diagnosis of EoE is dependent on the patient’s clinical manifestations, endoscopic assessment of the esophagus and histologic findings on esophageal mucosal biopsies.

### Clinical manifestations

Although the typical onset of EoE is in childhood, the disease can be found in all age groups and symptoms tend to vary depending on the age of presentation [13]. Clinical manifestations in infants and toddlers generally include vomiting, food refusal, choking with meals and, less commonly, failure to thrive. Predominant symptoms in school-aged children and adolescents include dysphagia (difficulty swallowing), food impactions, and choking/gagging with meals, particularly when comprised of foods with coarse textures. Other symptoms in this patient population include abdominal/chest pain, vomiting, and regurgitation. A careful history in children and adolescents with EoE reveals that they have learned to compensate for these symptoms by eating slowly, chewing excessively or taking small bites, drinking excessively with meals, lubricating meals inordinately with sauces, and avoiding specific food consistencies such as meats (or other foods with coarse textures) [14,15].

The predominant symptom in adults is dysphagia; however, intractable heartburn and food avoidance may also be present. Due to the long-standing inflammation and possible resultant scarring that has gone unrecognized, adults presenting with EoE tend to have more esophageal food impactions as well as other esophageal abnormalities such as Schatzki ring (a narrow ring of tissue located just above the junction of the esophagus and stomach), esophageal webs (small, thin growths of tissue that partially block the esophagus) and, in some cases, achalasia (an esophageal motility disorder characterized by difficulty swallowing and regurgitation). However, it is important to note that some patients with EoE are asymptomatic and suspicion of the disease is based upon incidental findings at endoscopy that is performed for other indications or upon evidence of food impaction in the absence of other symptoms.

Although many of these symptoms overlap with gastroesophageal reflux, the majority of patients with EoE exhibit a poor response to acid-suppression therapy (e.g., proton pump inhibitors [PPIs]), and up to 75% have a personal or family history of atopic disease (e.g., asthma, eczema, rhinitis) and environmental and/or food allergies [13]. Table 1 provides a brief summary of the clinical manifestations of EoE.

**Table 1:** Clinical manifestations of EoE.

Infants/Toddlers	Infants/Toddlers	Children	Adults
**Symptoms**	• Feeding aversion/intolerance • Vomiting • Food refusal • Choking with meals • Failure to thrive	• Dysphagia • Choking/gagging with coarse textures • Food impactions • Abdominal/chest pain • Vomiting/regurgitation	• Dysphagia (predominant) • Food impactions • Food avoidance • Intractable heartburn

**Response to acid-suppression therapy**	Poor	Poor	Poor

**Associated conditions**	• Food allergy • Atopic dermatitis	• Asthma • Allergic rhinitis • Food allergy	• History of atopy ο Asthma ο Allergic rhinitis

### Endoscopy

In order to help rule out GERD, an empiric 6- to 8-week trial of high-dose acid-suppression therapy is recommended before performing endoscopy in patients with suspected EoE. A barium swallow should also be considered to rule out severe small-calibre esophagus.

**Figure 1:**
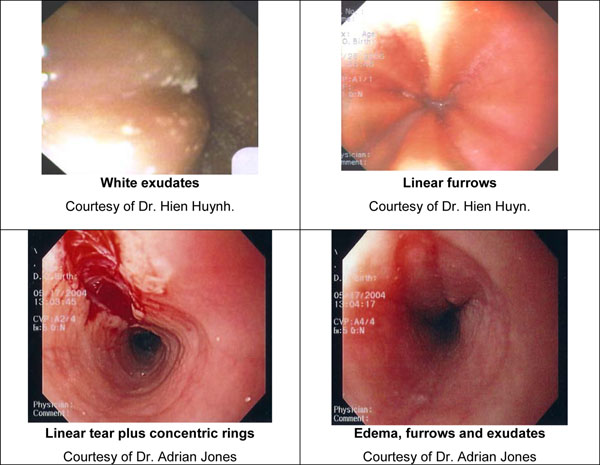
**Images of endoscopic features of EoE. A. White exudates** Courtesy of Dr. Hien Huynh. **B. Linear furrows** Courtesy of Dr. Hien Huyn. **C. Linear tear plus concentric rings** Courtesy of Dr. Adrian Jones. **D. Edema, furrows and exudates** Courtesy of Dr. Adrian Jones.

Although the endoscopic examination may be unremarkable, endoscopic features of EoE have been well-characterized and include: linear furrowing (ridges or furrows in the esophageal wall), concentric rings, white speckled exudates (eosinophilic abscesses), Schatzki ring, small-calibre esophagus, and linear superficial mucosal tears that occur after introduction of the endoscope [13]. Table 2 provides a more detailed description of each of these features. Images of exudates, linear furrows and tears are provided in Figure 1.

**Table 2:** Endoscopic features of EoE.

Endoscopic feature	Description
Linear furrowing	• Vertical esophageal lines or ridges in the esophageal wall

Concentric rings	• Multiple rings that may be fine, web-like or thickened (also termed the “corrugated” or “ringed” esophagus)

White speckled exudates	• Patches of whitish papules (1-2 mm in diameter) • Resembles esophageal candidiasis

Schatzki ring	• Narrow ring of tissue located just above the junction of the esophagus and stomach

Small-calibre esophagus	• Narrowed esophagus, with fixed internal diameter • Featureless, unchanging column • Poor expansion on air insufflation • Proximal and/or distal stenosis

Linear superficial mucosal tears	• Mucosal abrasions or shearing that occur upon minimal contact (e.g., after simple passage of a routine endoscope)

Although endoscopic findings are helpful in identifying patients with EoE [16], they are not diagnostic of the disease. Additionally, it is important to rule out esophageal candidiasis when white exudates are identified. As such, all patients with suspected EoE must undergo esophageal mucosal biopsies to confirm the diagnosis.

### Esophageal mucosal biopsies

Currently, endoscopic mucosal biopsy remains the most important diagnostic test for EoE. Biopsy specimens should be obtained regardless of the gross appearance of the mucosa, and specimens should be obtained from both the proximal and distal esophagus as well as areas revealing endoscopic abnormalities [2]. At least four biopsies are required to obtain a high sensitivity for the detection of EoE (5-6 biopsies are generally recommended) [13].

As discussed earlier, a definitive diagnosis of EoE is based on the presence of at least 15 eosinophils/HPF in the esophageal biopsies of patients who have normal pH studies or are refractory to acid-suppression therapy (i.e., to rule out GERD). GERD can increase eosinophilic infiltration in the distal esophagus, however, eosinophils associated with GERD generally occur at a lower density (i.e., <10/HPF) [1,17].

### Allergy assessment

A thorough personal and family history of other atopic conditions is recommended in all patients with EoE. Testing for allergic sensitization may be considered, with skin prick testing or blood testing for allergen-specific IgE, and potentially with atopy patch testing. However, it is important to remember that current approaches may not be able to accurately identify EoE triggers. The role of specific evaluation for allergic triggers in EoE remains in evolution, rendering it difficult to make standard recommendations.

Approximately two-thirds of patients with EoE have positive skin tests to at least one food allergen, most commonly dairy, eggs, wheat, soy and peanuts [2]. Unfortunately, the positive and negative predictive values for these tests have not been well established in this condition.

Atopy patch testing is a newer approach being used for the potential identification of non-IgE (cell-mediated) reactions. It is similar to patch testing for contact dermatitis and involves placing a small quantity of an allergen directly on the skin and then examining for a local, delayed reaction after a set time. Although atopy patch testing appears promising for the identification of foods that may elicit non-IgE-mediated reactions, this type of testing has not yet been standardized, and the positive and negative predictive values remain unknown. More studies are needed to assess the reliability and validity of atopy patch testing before it can be recommended for routine use in the diagnosis and monitoring of EoE.

## Treatment

Treatment strategies available for EoE fall into three categories: (1) avoidance of triggers through dietary modification, (2) pharmacologic therapy (corticosteroids, leukotriene modifiers and biologics), and (3) mechanical dilatation of the esophagus. It is important to note, however, that most of the published studies examining these therapies are case series, and there has been limited testing of these regimens in randomized controlled trials.

### Dietary management

Three dietary approaches for the management of EoE have emerged over the past decade: (1) the elemental diet, (2) empiric dietary restrictions (also referred to as the empiric six-food elimination diet), and (3) targeted dietary restrictions. The elemental diet involves the removal of all sources of potentially allergenic protein from the patient’s diet through the use of an amino acid-based formula for nutritional support. Assuming there is a favorable clinical and histologic response, one new food per week is reintroduced in a sequential fashion, beginning with the least allergenic foods (fruits and vegetables) to the most highly allergenic (e.g., dairy, soy, egg, wheat, and peanuts). A repeat endoscopic assessment is performed after the reintroduction of every 3-5 foods to ensure that the inflammation has not recurred.

Although the elemental diet is associated with high rates of clinical and histologic improvement in both adults and children with EoE (i.e., 90%), symptoms often recur after normalization of the patient’s diet [12,18]. Furthermore, given the unpalatable taste of the formula, most patients require feeding by nasogastric tube which may lead to adherence issues and impaired quality of life, particularly in adolescents and adults.

Targeted and empiric dietary restrictions are often employed before considering an elemental diet. Targeted dietary restrictions involve the elimination of foods based on the results of skin prick and atopy patch testing. Although response rates noted with this approach are lower than those noted with the elemental diet, targeted dietary restrictions have still been shown to be effective in approximately 70-80% of patients and may result in better patient adherence [12]. However, clinically-irrelevant positive results and false negative results complicate this dietary approach and, therefore, further studies examining both the positive and negative predictive value of targeted dietary restrictions in EoE are necessary.

Rather than basing dietary elimination on skin prick testing and atopy patch testing, empiric dietary restrictions involve the elimination of the six most common allergic foods (regardless of the results of allergy testing): dairy, eggs, wheat, soy, peanuts, and fish/shellfish. Like targeted dietary restrictions, the empiric food elimination diet has been shown to be effective in approximately 75% of patients with EoE, and may also be associated with better patient adherence than the elemental diet [19].

With all dietary approaches, it remains unclear how long specific foods need to be avoided, and whether or not the patient’s diet can be normalized over time. Clearly, more studies on this approach are necessary, including an attempt to evaluate patient quality of life given the extensive dietary restrictions often required, including a great many “staple” foods. Furthermore, if several foods are to be eliminated simultaneously, enlisting the assistance of a dietitian may be beneficial, particularly in the pediatric population. This may help ensure nutritional requirements continue to be met in order to facilitate adequate growth and development.

### Pharmacologic management

Medical therapies for EoE include corticosteroids, leukotriene modifiers and biologic agents. Systemic (oral) corticosteroids were one of the first treatment options shown to be effective in patients with EoE. Both clinical and histologic improvement have been noted in approximately 95% of EoE patients using systemic corticosteroids; however, upon discontinuation of therapy, 90% of patients experience a recurrence in symptoms [20]. Furthermore, given that prolonged use of systemic corticosteroids are associated with well-known and potentially serious adverse effects, their long-term use is not recommended. Systemic corticosteroids should be reserved for emergent cases such as patients with dysphagia requiring hospitalization or patients experiencing significant weight loss or dehydration due to swallowing difficulties [2].

Given their substantially better safety profile, topical corticosteroids delivered to the esophagus have become the mainstay of pharmacotherapy for patients with EoE. Both swallowed fluticasone propionate (500-1000 µg/day) and oral viscous (thick) budesonide (500-1000 µg/day) have been shown to be effective in the management of EoE. Fluticasone propionate is delivered via a pressurized metered dose inhaler (pMDI) that is activated into the mouth (without inhaling and without a spacer device) and swallowed. Budesonide is administered orally after the contents of a vial used for nebulization are mixed with an artificial sweetener to increase the viscosity (thickness) of the solution which, theoretically, slows its transit over the esophageal lining [13].

Randomized clinical trials of topical fluticasone propionate therapy have shown both histologic and symptomatic improvements in 50-80% of patients with EoE [21,22]. The most frequent complications noted with topical fluticasone propionate are superficial oropharyngeal and esophageal candidiasis. Although not as well studied as topical fluticasone propionate, oral viscous budesonide also appears to lead to similar clinical and histologic response rates in pediatric patients with EoE [23,24]. Oral budesonide has been associated with a lower risk of developing esophageal candidiasis, and given the viscosity of the solution, may provide better delivery to the esophageal surface than swallowed fluticasone. However, budesonide has a slightly higher systemic bioavailability than oral fluticasone propionate and, therefore, may potentially be associated with more systemic effects.

Patients using topical corticosteroids for EoE should be advised not to eat, drink, or rinse their mouth for 20 to 30 minutes after using the medication [13]. After 6-8 weeks of topical therapy, patients should undergo repeat endoscopic assessment to ensure histologic response to therapy. If a therapeutic response is confirmed, treatment should be reduced to the lowest effective dose with appropriate follow up. It is important to note that symptoms and pathological changes often recur after topical corticosteroid therapy is discontinued. Therefore, many patients with EoE will require long-term treatment.

Since inflammatory mediators such as leukotrienes have been theorized to play a role in the esophageal inflammation noted in patients with EoE, leukotriene modifiers may be of value for the management of EoE [2]. A small study of 8 patients with EoE examined the efficacy of the leukotriene receptor antagonist, montelukast, and found a significant improvement in symptoms in the majority of subjects, but no improvement in histology [25]. Given that montelukast is generally well tolerated and potentially useful for the management of other atopic diseases such as asthma, it may be a therapeutic option to consider in patients with EoE and concurrent atopic conditions.

Given that both IL-5 and IgE appear to play a role in the pathogenesis of EoE, humanized monoclonal antibodies against IL-5 (reslizumab, mepolizumab) and IgE (omalizumab) may also be potential therapeutic options for the disease. Results from small case series using anti-IL-5 antibodies in patients with EoE suggest that these biologics are well tolerated and may improve clinical symptoms, histology and quality of life [26]. Two large pediatric trials are currently underway to further examine the efficacy and safety of anti-IL-5 antibodies in the management of EoE.

The anti-IgE antibody omalizumab is used for the management of severe atopic asthma and allergic rhinitis. Since omalizumab has been shown to lower eosinophil counts in the blood and lungs of asthmatic subjects [27], it may be a potential therapeutic approach for EoE. Although no clinical trials of omalizumab in patients with EoE have been conducted, anecdotal reports suggest that this may be a promising treatment option.

### Endoscopic dilatation

Esophageal endoscopic dilatation is a treatment modality most commonly used in adults with established esophageal strictures. Although dilatation is effective for relieving dysphagia, it does not address the underlying inflammation and, therefore, the majority of patients undergoing this procedure develop recurrent symptoms within 3-8 months [4,28-30]. Furthermore, in patients with EoE, endoscopic dilatation has been associated with extensive mucosal tearing and perforation. Therefore, dilatation is generally reserved for patients with symptomatic strictures that persist after a trial of pharmacological or dietary therapy [2,7,13].

**Figure 2:**
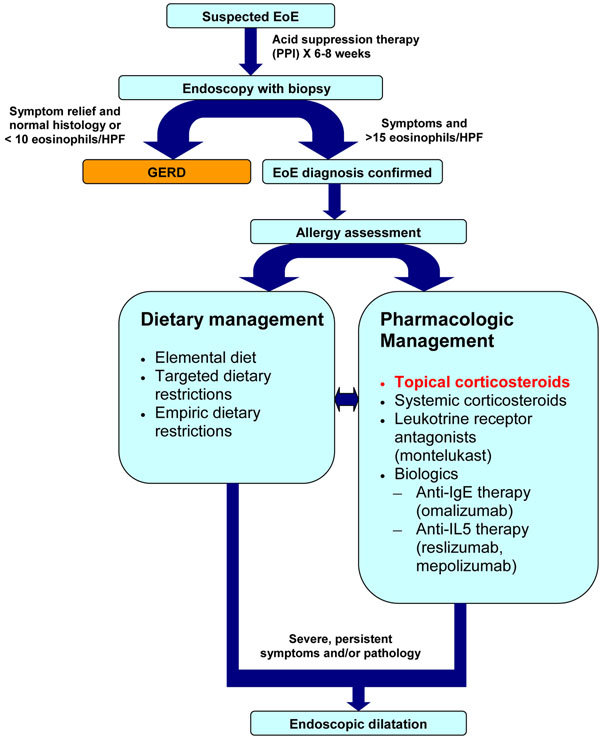
**Proposed algorithm for the diagnosis and management of EoE. ***EoE: eosinophilic esophagitis; PPI: proton pump inhibitor; GERD: gastrointestinal reflux disease; HPF: high power field; IgE: immunoglobulin E; IL5: interleukin 5*

A proposed algorithm for the diagnosis and management of EoE is shown in Figure 2.

## Prognosis

The long-term prognosis for patients with EoE is unknown. Some patients may follow a “waxing and waning” course characterized by symptomatic episodes followed by periods of remission. There have also been reports of apparent, spontaneous disease remission in some patients; however, the risk of recurrence in these patients is unknown. It is possible that long-standing, untreated disease may result in esophageal remodeling, leading to strictures, Schatzki ring and, eventually, achalasia. Currently, it is still unclear if dietary or medical therapy modifies the natural history of the disease [1].

## Conclusions

EoE is an evolving condition that requires further study to better understand the mechanisms of disease development and tissue injury, the natural history, and optimal management. Although clearly an atopic condition, the role of specific allergic triggers in EoE remains unclear. However, as our understanding surrounding EoE improves, so will strategies for the diagnosis and treatment of the condition.

## Key take-home messages

• EoE is an atopic condition of the esophagus that has become increasingly recognized over the last decade.

• Endoscopic mucosal biopsy revealing >15 eosinophils/HPF in one or more specimens remains the most important diagnostic test for EoE.

• Patients with EoE should be referred to an allergist to help identify potential triggers, optimize treatment, and manage concurrent atopic conditions.

• The elemental diet, empiric dietary restrictions and targeted dietary restrictions are associated with high rates of clinical and histologic improvement in patients with EoE.

• Topical corticosteroids delivered to the esophagus are the mainstay of pharmacotherapy for patients with EoE.

• Esophageal endoscopic dilatation should be reserved for patients with symptomatic strictures that persist after a trial of pharmacological or dietary therapy.

## Competing interests

Dr. Stuart Carr has received consulting fees and honoraria for continuing education from GlaxoSmithKline, Graceway, Merck, Novartis, Nycomed, and Paladin. He is a local principal investigator for a Ception-sponsored study of reslizumab, a novel biological therapeutic agent for eosinophilic esophagitis. Dr. Carr did not receive any incentive or funding for the preparation or review of the manuscript.

Dr. Wade Watson is a co-chief editor of *Allergy*, *Asthma & Clinical Immunology*. He has received consulting fees and honoraria for continuing education from AstraZeneca, GlaxoSmithKline, King Pharma, Merck Frosst, and Nycomed.
